# Phospholipase Cε Modulates Rap1 Activity and the Endothelial Barrier

**DOI:** 10.1371/journal.pone.0162338

**Published:** 2016-09-09

**Authors:** Peter V. DiStefano, Alan V. Smrcka, Angela J. Glading

**Affiliations:** Department of Pharmacology and Physiology, University of Rochester, Rochester, New York, 14642, United States of America; BloodCenter of Wisconsin, UNITED STATES

## Abstract

The phosphoinositide-specific phospholipase C, PLCε, is a unique signaling protein with known roles in regulating cardiac myocyte growth, astrocyte inflammatory signaling, and tumor formation. PLCε is also expressed in endothelial cells, however its role in endothelial regulation is not fully established. We show that endothelial cells of multiple origins, including human pulmonary artery (HPAEC), human umbilical vein (HUVEC), and immortalized brain microvascular (hCMEC/D3) endothelial cells, express PLCε. Knockdown of PLCε in arterial endothelial monolayers decreased the effectiveness of the endothelial barrier. Concomitantly, RhoA activity and stress fiber formation were increased. PLCε-deficient arterial endothelial cells also exhibited decreased Rap1-GTP levels, which could be restored by activation of the Rap1 GEF, Epac, to rescue the increase in monolayer leak. Reintroduction of PLCε rescued monolayer leak with both the CDC25 GEF domain and the lipase domain of PLCε required to fully activate Rap1 and to rescue endothelial barrier function. Finally, we demonstrate that the barrier promoting effects PLCε are dependent on Rap1 signaling through the Rap1 effector, KRIT1, which we have previously shown is vital for maintaining endothelial barrier stability. Thus we have described a novel role for PLCε PIP_2_ hydrolytic and Rap GEF activities in arterial endothelial cells, where PLCε-dependent activation of Rap1/KRIT1 signaling promotes endothelial barrier stability.

## Introduction

Phospholipase C (PLC) family members are common mediators of signal transduction in mammalian cells. Upon activation by growth factor receptors or G-protein coupled receptors, PLC cleaves phosphatidylinositol 4,5-bisphosphate (PIP_2_), into inositol trisphosphate (IP_3_) and diacylglycerol (DAG), which then mediate pleiotropic downstream effects on cell migration, proliferation, and cell contractility. There are six different sub-types of PLC, which all contain a conserved catalytic region, EF hand, and phospholipid binding domain[1,2]. PLCε is unique among the PLC family as it possesses an N-terminal CDC25 GEF domain and two C-terminal Ras association (RA) domains, RA1 and RA2, allowing it to act as regulator and effector of Ras subfamily small GTPase signaling[1]. PLCε has been shown to increase the exchange of GDP for GTP in Rap1 via its CDC25 GEF domain [1,3]. Subsequently, Oestreich et al showed that basal Rap1 activity was diminished in PLCε knockout hearts compared to wild type[4]. The RA2 domain, on the other hand, binds Rap1 and Ras and allows these proteins to stimulate the lipase activity of PLCε [5–9]. The presence of these small GTPase signaling domains in PLCε suggests that it acts as a specific link between GF/GPC receptor activity and Ras family GTPases. However, the role of PLCε in regulating small GTPase signaling has only been explored in a few cellular contexts.

Rap1 GTPase is a master regulator of cell adhesion, including both cell-matrix and cell-cell adhesion. Its activity is tightly controlled by the combined action of Rap1 exchange factors (GEFs) and activating proteins (GAPs), which promote or reduce the amount of active, GTP bound, Rap1. In cultured endothelial cells, activation of Rap1 is essential for the formation of cell-cell junctions and baseline barrier function[10], and decreasing Rap1 activity via knockdown of PDZ-GEF or by expressing Rap1-GAP decreases endothelial barrier integrity[11,12]. Deletion of both Rap1 isoforms in vivo leads to hemorrhage, vascular rupture, and microvessel dilation, though with incomplete penetrance of the phenotype[13]. These data indicate that Rap1 activity is a major regulatory focus for endothelial barrier function, and suggest that the consistent maintenance of basal Rap1 activity may be a critical component of vascular homeostasis.

Rap1 is known to modulate endothelial cell-cell contacts via interactions with a number of proteins. Of these, it has recently emerged that Krev Interaction Trapped 1 (KRIT1) is essential for the ability of active Rap1 to stabilize endothelial cell-cell junctions, both at a basal level and downstream of a number of stimuli[12,14,15]. KRIT1 is a scaffold protein that co-localizes with β-catenin at areas of cell-cell contact[12]. Activation of Rap1 promotes KRIT1 membrane localization and junctional stability that is dependent on KRIT1-Rap1 binding[14]. KRIT1 expression is required for the ability of Rap1 to limit thrombin-induced leak [12], and reduced KRIT1 expression in vitro and in vivo destabilizes the endothelial barrier[12,16–18]. Another recent study has shown that the barrier-promoting properties of prostacyclin, which are known to occur via Rap1 activation, requires the Rap1-binding capacity of KRIT1[15]. This evidence suggests that the Rap1-KRIT1 signaling axis is a common component of endothelial barrier stabilization pathways. However, it is not known whether PLCε, which can regulate Rap1 activity, can effectively regulate endothelial barrier stability, nor whether this requires the presence of KRIT1.

In this study we show that PLCε is expressed abundantly in primary endothelial cells and endothelial cell lines, and that loss of PLCε protein expression is sufficient to reduce the integrity of an endothelial monolayer. Reduced endothelial barrier function corresponded with increased Rho-GTP levels and cytoplasmic stress fiber formation, and with decreased Rap1 activity. Structure function experiments revealed that a CDC25-deficient PLCε was unable to rescue the effect of PLCε depletion on monolayer integrity. We were surprised to find that a mutant PLCε lacking lipase activity also could not fully rescue endothelial monolayer integrity, and exhibited a moderate inhibitory effect on Rap1-GTP levels. Finally, we demonstrate that loss of the Rap1 effector, KRIT1, does not increase monolayer leak in PLCε-deficient cells, suggesting that PLCε and KRIT1 are acting in the same pathway. Together, our findings strongly suggest that PLCε promotes endothelial barrier integrity by maintaining basal levels of Rap1-GTP through its lipase and CDC25 GEF domain, feasibly through promoting KRIT1 membrane localization and subsequent stabilization of endothelial cell-cell contact.

## Experimental procedures

### Ethics Statement

The isolation of neonatal rat ventricular fibroblasts was carried out in accordance with the recommendations of the NIH Guide for the Care and Use of Laboratory Animal and approved by the University Committee on Animal Resources of the University of Rochester (Protocol number 2007-137R issued to Alan V. Smrcka).

### Cell Culture and Transfection

HPAEC (Invitrogen, Carlsbad, CA), hCMEC/D3 (gift of Dr. Babette Wesker, Weil Cornell Medical College), and HUVEC (Lonza, Basel, Switzerland) were cultured in 1:1 Dulbecco’s modified Eagle’s medium (DMEM): F/12, supplemented with 5% fetal bovine serum (FBS), 1% endothelial cell growth supplement (ECGS, ScienCell, Carlsbad, CA), 1% antimyotic/antibiotic solution (Gibco/Invitrogen), and 50μM heparin (Calbiochem, La Jolla, CA), at 37°C with 5% CO_2_. HPAEC were grown on 2μg/cm^2^ gelatin coated tissue culture plates and only passages 3–6 were used in experiments. Neonatal rat ventricular fibroblasts were isolated from wildtype Sprague-Dawley rats and lysed in Laemelli buffer. HPAEC were transfected with 30ng siRNA using the HiPerfect transfection reagent (Qiagen, Valencia, CA) as reported previously [12,19]. Transfection efficiencies ranged from 80–95% based on transfection of fluorescently labeled siRNAs (data not shown).

### Isolation of Neonatal Rat Ventricular Fibroblasts

Neonatal Rat Ventricular Fibroblasts were isolated from P2 Sprague-Dawley rat pups (Harlan Sprague-Dawley Inc./Envigo, Indianapolis, Indiana) that were sacrificed by immersion in 70% ethanol, followed by decapitation. Briefly, the hearts were excised and the ventricles were separated and minced in Hank’s buffered salt solution (HBSS) containing CaCl_2_ and MgCl_2_ (Invitrogen) supplemented with 0.1% BSA (Sigma) and penicillin/streptomycin (Invitrogen), followed by three rounds of digestion with 250U/mg collagenase Type 2 (Worthington, Lakewood, NJ). The digestion mixture was spun down and the cell containing pellet was resuspended in DMEM (Lonza) supplemented with 2mM L-glutamine (Invitrogen), 2% penicillin/streptomycin, 200 ng/ml Vitamin B12 (Sigma), and 10% FBS, and pre-plated on an uncoated tissue culture dish for 1 hour. Myocytes were washed off the plate and the adhered fibroblasts were expanded for 3 days, then lysed in Laemelli buffer for Western blot.

### siRNA and Plasmids

Non-targeting negative control siRNA #1 and anti-KRIT1 siRNA (AM16708, Ambion/Invitrogen) were used as reported previously[12,19]. Knockdown efficiency was measured in each experiment by protein detection of KRIT1, as indicated in the appropriate figures.

### Adenovirus Infection

To generate the PLCε ΔCDC25/lipase dead clone (ΔCDC25/LD), the N-terminal cDNA of PLCε lipase dead was digested with NheI and NdeI and replaced with the corresponding region of PLCε ΔCDC25 in pShuttle-CMV. The identity of the clone was confirmed by restriction digestion, sequencing, and Western blotting. Adenovirus for this construct was generated in the same manner used to generate the PLCε ΔCDC25, PLCε lipase dead (LD), PLCε K2150E, PLCε siRNA, random siRNA, and PLCε siRNA resistant (WT) adenoviruses as reported previously[4,5,20,21]. HPAEC were infected with 60 m.o.i of random, PLCε siRNA, or siRNA resistant PLCε or 120 m.o.i of PLCε ΔCDC25, PLCε lipase dead, PLCε K2150E, or PLCε ΔCDC25/lipase dead for 4 hours followed by a medium change.

### Immunoprecipitation and Western Blotting

Cells were treated with or without 1μM 8-pCPT-2′-O-Me-cAMP-AM (Tocris/R&D Biosystems) as indicated in the figure legends. Lysates were prepared as reported previously[12]. KRIT1 was immunoprecipitated using 2μg monoclonal anti-KRIT1 (EMD Millipore, Darmstadt, Germany). Lysates were probed with polyclonal rabbit anti-KRIT1 Δ6832 (Ginsberg Lab, UCSD) at a dilution of 1:1000. Control lysates were immunoprecipitated with mouse non-immune IgG (Santa Cruz, Dallas, TX). Antibodies used for Western blot were rabbit anti-actin (Sigma, St. Louis, MO), rabbit anti-GAPDH (Santa Cruz), rabbit anti-PLCε (Smrcka Lab, University of Rochester), and rabbit anti-VE cadherin (Cayman Chemical, Ann Arbor, MI). The secondary antibodies used were goat anti-rabbit Dylight-680 (Thermo Fisher Scientific) and goat-anti rabbit 800 (Fisher). An Odyssey Infrared Imaging System (LI-COR Biosciences, Lincoln, NE) was used to image membranes and for densitometry.

### Endothelial Monolayer Leak Assay

HPAECs were plated onto 3μm pore polyester transwell filters (Corning Life Sciences, Corning, NY) coated with 10μg/ml human plasma fibronectin (gift from Dr. Denise Hocking, University of Rochester). Cells were infected with adenovirus 24 hours after plating and grown for 72 hours at 37°C to full confluence. Cells were then incubated in DMEM with 0.5% FBS for 2 hours, then treated with or without 8-pCPT-2′-O-Me-cAMP-AM (1μM) for 30 minutes. Horseradish peroxidase (HRP, 1.5μg/ml, Sigma) was added to upper wells, and the plates were then incubated for an additional 2 hours at 37°C. The HRP content of the lower chamber medium was then measured using a 3,3’, 5,5’-tetramethylbenzidine (TMB, eBioscience, San Diego, CA) colorimetric assay. Briefly, 10μl samples of the lower chamber medium were transferred in triplicate to a 96well plate. 100μl of TMB was added to each sample and the reaction was allowed to proceed for 1 min. A standard curve of HRP from 0 to 0.5μg/ml was run alongside experimental samples. The reaction was stopped by adding 100μl 1N HCL to each well. Absorbance at 450nm was acquired and raw absorbance values were converted into concentrations using the standard curve. After samples were removed, the transwell filters were fixed in 4% formaldehyde (Fisher), stained with 0.25% Coomassie blue (Bio-Rad, Hercules, CA), and examined to confirm the integrity of each cell monolayer. Alternatively, 1 mg/ml fluorosecein isothiocyanate-dextran of 4, 70, or 150 kDa molecular weight (FITC-dextran, Sigma) was added to the top chamber ± 4U/ml thrombin (Sigma). At the reported time intervals, 50 μl samples were taken from the bottom chamber and fluorescence was measured at excitation 485 nm/emission 520 nm on a Synergy H4 hybrid plate reader (Biotek, Winooski, VT). Data were analyzed using one-way ANOVA with Tukey’s post-hoc testing.

### Immunofluorescence

Cells were infected 24 hours after plating on 10μg/ml fibronectin coated glass coverslips and allowed to grow for an additional 48 hours to confluence, then treated with or without 1μM 8-pCPT-2′-O-Me-cAMP-AM. Cells were fixed with 4% formaldehyde for 20 minutes, permeabilized for 5 minutes with 0.2% Triton-X-100, then blocked with 10% normal goat serum for 1 hour. Cells were then incubated with 1:300 rabbit anti-VE-cadherin (Cayman Chemical), followed by incubation with 1:500 goat anti-rabbit IgG 488 (Invitrogen). Cells were counterstained with 1 μg/ml Hoechst (Invitrogen) for 5min prior to mounting. VE-cadherin staining was quantified by measuring the width of half-maximal VE-cadherin staining intensity at cell borders using ImageJ.

### F-actin quantitation

Cells were incubated with 14μM rhodamine-phallodin (Cytoskeleton, Denver, CO) for 30 minutes on ice then counterstained with 1μg/ml Hoechst. Images were captured at room temperature using Metamorph software (Molecular Devices, Sunnyvale, CA) with an UPLSAPO 20x (n.a. 0.75) objective on an Olympus IX81 microscope, and an ORCA-ER digital camera (Hamamatsu, Hamamatsu City, Japan). Images were imported into ImageJ for further analysis. Cytoplasmic F-actin fluorescence intensity was calculated along a line scan drawn perpendicular to the main cell axis as reported in (16). Cortical actin intensity along the cell edge was calculated in a similar manner, with the line drawn perpendicular to the cell edge and to the main cell axis, incorporating the junction between two cells but no more than 15% of the width of the cytoplasm. Intensity values were obtained by integrating the area under the curve of the intensity histogram generated by the line scan. The area calculated was then divided by length of the line scan, yielding an average intensity value. Data were analyzed using one-way ANOVA with Tukey’s post-hoc testing.

### Small GTPase Pulldowns

After stimulation with or without 1μM 8-pCPT-2′-O-Me-cAMP-AM, cells were lysed in MgALB lysis buffer (50mM Tris HCl pH 7.5, 200mM NaCl, 2mM MgCl_2_, 10% glycerol, 1% NP-40) or 1X RIPA lysis buffer, for Rap1 and RhoA pulldowns respectively. Lysates were clarified at 14,000 RPM for 5 minutes followed by incubation with 20μg GST RalGDS-RBD or GST rhotekin-RBD immobilized on GSH-sepharose for 45 minutes at 4°C. Afterwards, the sepharose beads were washed 3X with lysis buffer, re-suspended and boiled in 2X reducing Laemelli buffer, and centrifuged. Supernatant was then loaded on SDS PAGE gel for Western blot analysis. Mouse anti-Rap1 (Santa Cruz) and mouse anti-RhoA (Cytoskeleton) were used at 1:500 followed by incubation with a 1:5000 dilution of HRP conjugated goat anti-mouse IgG. Blots were developed using CL-XPosure film (Thermo Fisher Scientific) and analyzed using Image Studio Lite software (LI-COR Biosciences).

### Statistics

Statistical analysis (i.e. one-way ANOVA with appropriate post-hoc testing) was performed using PRISM software (version 4.0, GraphPad Software Inc., La Jolla, CA). Significance was set at a = 0.05.

## Results

### PLCε is highly expressed in the endothelium and limits monolayer leak

**Fig 1:**
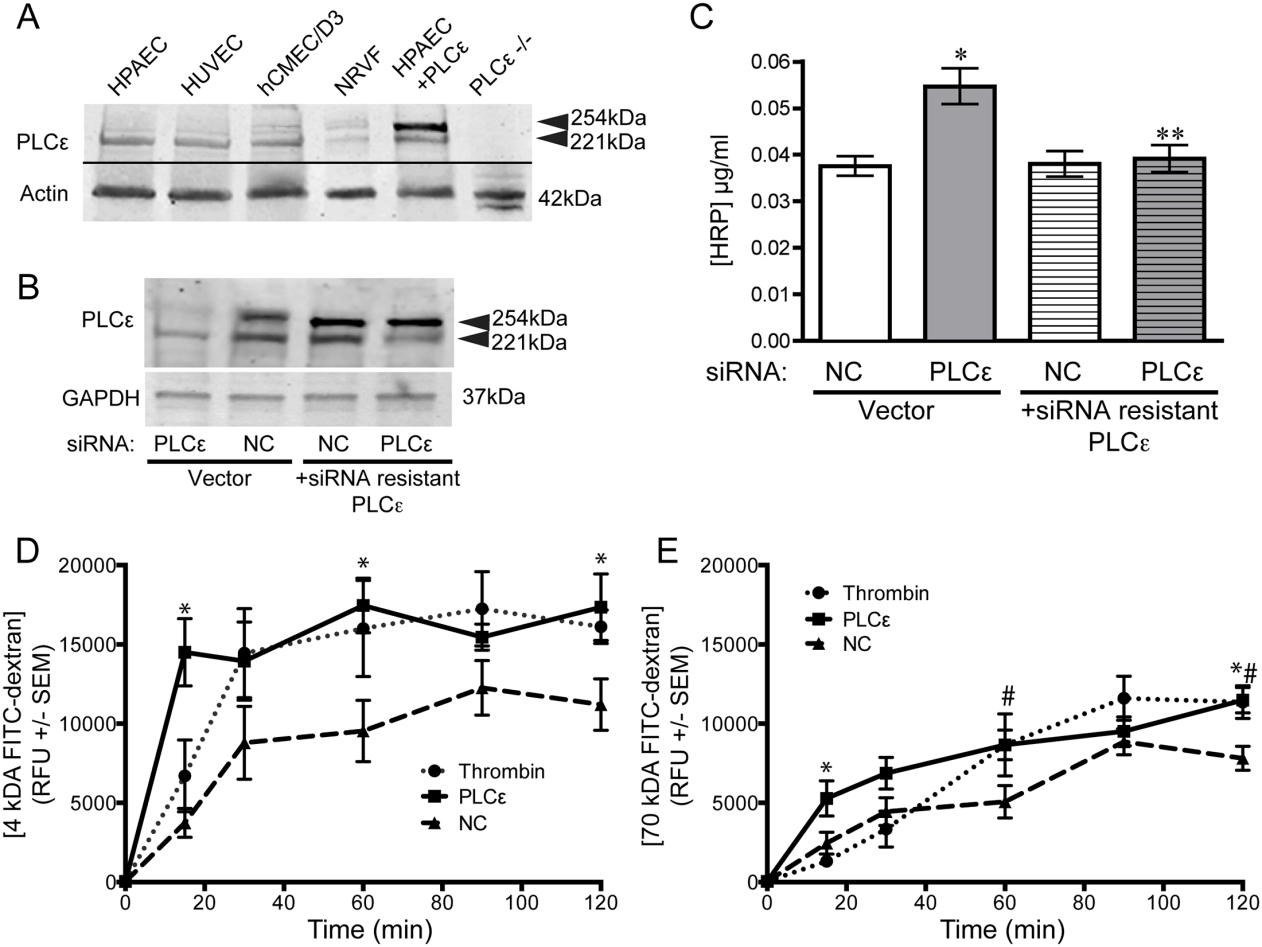
PLCε is expressed in endothelial cells and limits monolayer leak. **A**) PLCε expression in equivalent amounts of endothelial, NRVF, and heart lysate. HPAEC+ PLCε, HPAEC over-expressing PLCε; PLCε -/-, PLCε knockout heart lysate. Blots are representative, n = 3. **B**) PLCε expression in HPAEC infected with random siRNA (negative control, NC) or PLCε siRNA ± siRNA resistant PLCε. Blots are representative, n = 3. **C**) HRP leak through a HPAEC monolayer infected with negative control (NC) or PLCε siRNA ± siRNA resistant PLCε. Data shown are mean HRP concentration, ± SEM. n = 6, p<0.0001 by ANOVA, *p<0.001 by post-hoc test vs. NC infected cells and **p<0.001 vs. PLCε siRNA infected cells. **D**) Leak of 4 kDa FITC-dextran through an HPAEC monolayer infected with negative control (NC) ± 4U/ml thrombin or PLCε siRNA. Data shown are relative fluorescence units (RFU), ± SEM, n = 8. *p<0.05 PLCε siRNA vs. NC by 2-way ANOVA. **E**) Leak of 70 kDa FITC-dextran through an HPAEC monolayer infected with negative control (NC) ± 4U/ml thrombin or PLCε siRNA. Data shown are RFU ± SEM, n = 8. *p<0.05 PLCε siRNA vs. NC, #p<0.05 thrombin vs. NC, by 2-way ANOVA.

PLCε has been reported to be expressed endogenously in a multitude of cell types including Rat-1 fibroblasts, astrocytes, pancreatic beta cells, mouse ventricular myocytes, and neonatal rat ventricular myocytes [22–25]. Expression of PLCε mRNA has been reported in freshly isolated murine endothelial cells but not HUVEC[26,27]. We detected high levels of PLCε protein in endothelial cells from multiple origins, including HPAEC, HUVEC, and immortalized brain microvascular endothelial cells (hCMEC/D3, Fig 1A). The anti-PLCε antibody, which recognizes the RA2 domain of PLCε[25], detected a doublet at approximately 254 and 221 kDa (Fig 1A), corresponding to the splice variants of PLCε, PLCε1a and PLCε1b, which differ in the number of amino acids N-terminal to the CDC25 domain[25,28]. The molecular weight of the bands matched those seen in neonatal rat ventricular fibroblasts (NRVF) and the upper band was significantly enhanced in HPAEC over-expressing PLCε1a. No bands were detected in PLCε knockout heart lysate (Fig 1A).

To examine the role of PLCε in endothelial cells, we knocked down PLCε expression using virally delivered anti-PLCε siRNA, which targets the RA1 domain[21,24]. Western blotting confirmed that 60 m.o.i. reduced PLCε1a protein expression by 70% and PLCε1b expression by 50% (Fig 1B). We could restore PLCε expression by co-infection with siRNA resistant wild type PLCε1a (Fig 1B). We then examined a key endothelial phenotype, barrier stability, in PLCε depleted cells, using a transwell leak assay. Knockdown of PLCε increased the leak of horseradish peroxidase (HRP, ~30kDa) 1.7-fold over random-siRNA infected cells. siRNA specificity was confirmed by rescuing monolayer barrier function via re-expression of siRNA resistant wild type PLCε (Fig 1C). PLCε knockdown also increased the diffusion of both 4- and 70kDa FITC-labeled dextran through the monolayer compared to control siRNA infected cells. As shown in Fig 1D, 4kDa FITC-dextran moves across the monolayer rapidly in both PLCε siRNA and thrombin treated cells, reaching equilibrium within 30 minutes. Comparatively, flux of 70kDa FITC-dextran occurs at a much slower rate, with equilibrium not reached after 2hr. Notably, the rate of flux for both dextran sizes was similar in PLCε siRNA treated cells and cells treated with thrombin. Indeed, both thrombin and PLCε siRNA were not able to significantly increase the flux of 150kDa FITC-dextran (S1 Fig), suggesting the ‘pore size’ generated by knockdown of PLCε has a finite range.

### Depletion of PLCε activates RhoA and leads to cytoskeletal rearrangement in HPAEC

**Fig 2:**
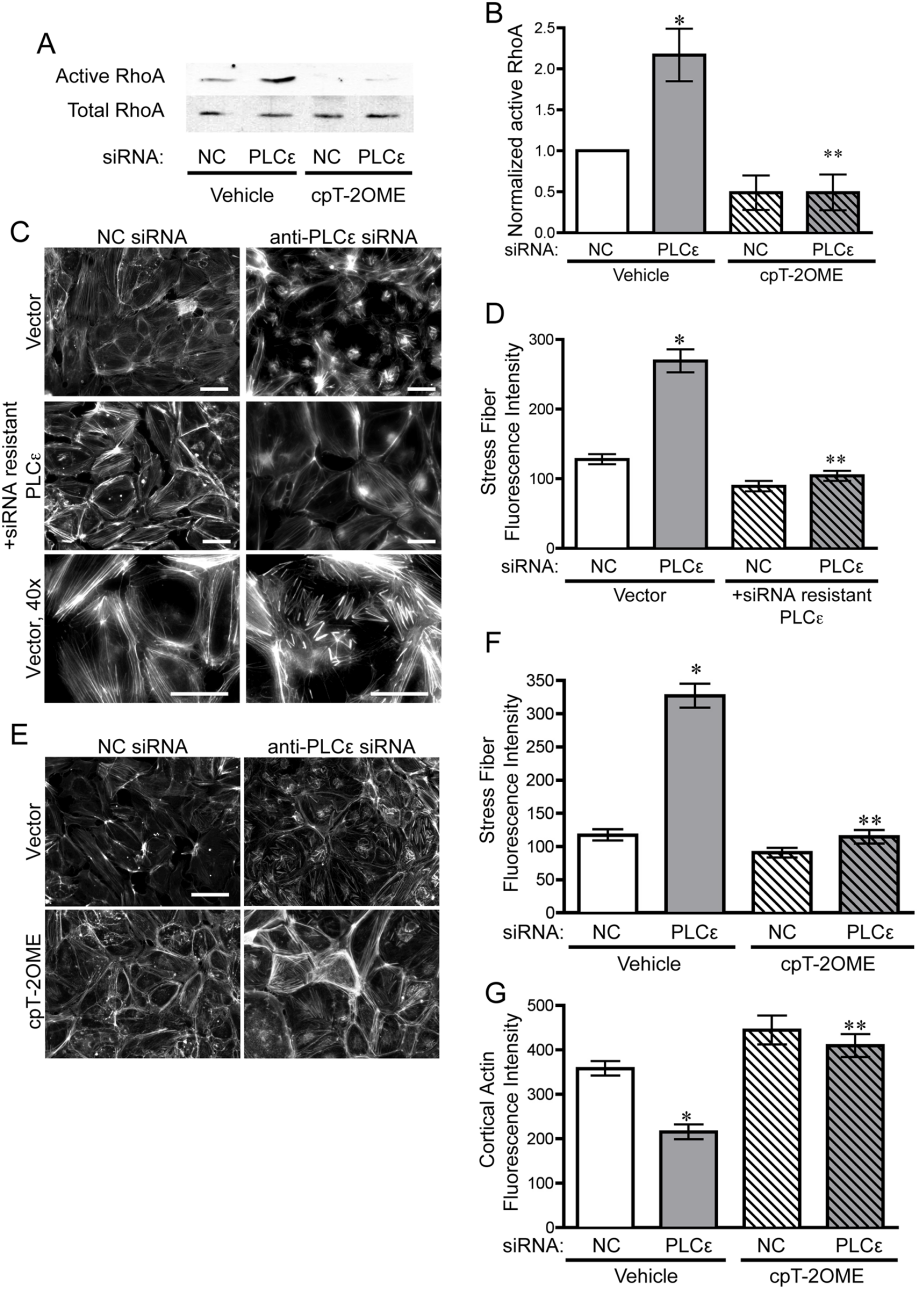
PLCε depletion leads to increased RhoA activity and stress fiber formation. **A**) Active RhoA pulldown assay from lysates infected with negative control (NC) or PLCε siRNA ± 1μM 8-pCPT-2′-O-Me-cAMP-AM (cPT-2OMe). Blots are representative, n = 4. **B**) Densitometric quantification of blots in (A). Data shown are active RhoA normalized to total RhoA ± SEM, n = 4, p = 0.0005 by ANOVA, *p<0.05 vs. NC infected cells, and **p<0.001 vs. PLCε siRNA infected cells by Tukey’s Multiple Comparison Test. All blots used for this quantification can be found in S5 Fig. **C**) Rhodamine-phalloidin stained HPAEC infected with NC or PLCε siRNA ± siRNA resistant PLCε. Scale bar = 50μm. Images are representative of 3 separate experiments. **D**) Fluorescent intensity quantification of (C). n≥40 cells from 10 fields of view, p<0.0001 by ANOVA, *p<0.001 by post-hoc test vs. NC infected cells. ** p<0.001 vs. PLCε siRNA infected cells. **E**) Epifluorescence images of rhodamine-phalloidin stained HPAEC infected with negative control (NC) or PLCε siRNA ± 1μM 8-pCPT-2′-O-Me-cAMP-AM. **F**) Fluorescent intensity quantification of cytoplasmic stress fibers (E). n≥40 cells from 10 fields of view, p<0.0001 by ANOVA, *p<0.001 by post-hoc test vs. NC infected cells. ** p<0.001 vs. PLCε siRNA infected cells. **G**) Fluorescent intensity quantification of cortical actin in (E). n≥40 cells from 10 fields of view, p<0.0001 by ANOVA, *p<0.001 by post-hoc test vs. NC infected cells. ** p<0.001 vs. PLCε siRNA infected cells.

Endothelial monolayer leak is commonly associated with an increase in the activity of the small GTPase, RhoA. RhoA-GTP activates Rho-associated protein kinase, which subsequently phosphorylates myosin light chain to increase actin-myosin contraction and increase the formation of transverse cellular actin bundles (stress fibers, [29]. The subsequent increase in cellular tension exerts a negative influence on the stability of cell-cell contacts. RhoA-mediated increased cellular contractility occurs under many conditions that increase endothelial leak. Therefore, we examined RhoA activity and the morphology of the actin cytoskeleton in PLCε-deficient cells. RhoA activity increased approximately two-fold in PLCε-deficient cells over control cells (Fig 2A and 2B). While large transverse stress fibers are a hallmark of RhoA activation[30], when we examined the morphology of the actin cytoskeleton, a large proportion of PLCε depleted cells exhibited extremely short, stubby perinuclear stress fibers that are relatively atypical of activation of RhoA (Fig 2C). Quantitation of the distribution of actin fibers in the cells, however, clearly shows an increase in cytoplasmic actin staining in the endothelial monolayer, indicative of an increase in cellular contractility, as well as a decrease in cortical actin intensity (Fig 2D–2G).

Next, we asked whether increasing Rap1 activity through the Rap1 GEF, Epac, which acts independently of PLCε, could reverse the increase in RhoA activity. We and others have demonstrated that RhoA activation can inhibited by active Rap1[10,12], suggesting that the activation of RhoA in PLCε-deficient cells could be due to a loss of Rap1 activity. Treatment of PLCε depleted cells with 8-pCPT-2′-O-Me-cAMP-AM, a potent and specific Epac activator[31], successfully reduced the amount of active RhoA down to control levels (Fig 2A), and reversed the stress fiber phenotype (Fig 2E–2G), indicating that loss of Rap1 activation could account for the increase in RhoA activity.

### Reduced active Rap1 levels following depletion of PLCε is responsible for monolayer leakiness

**Fig 3:**
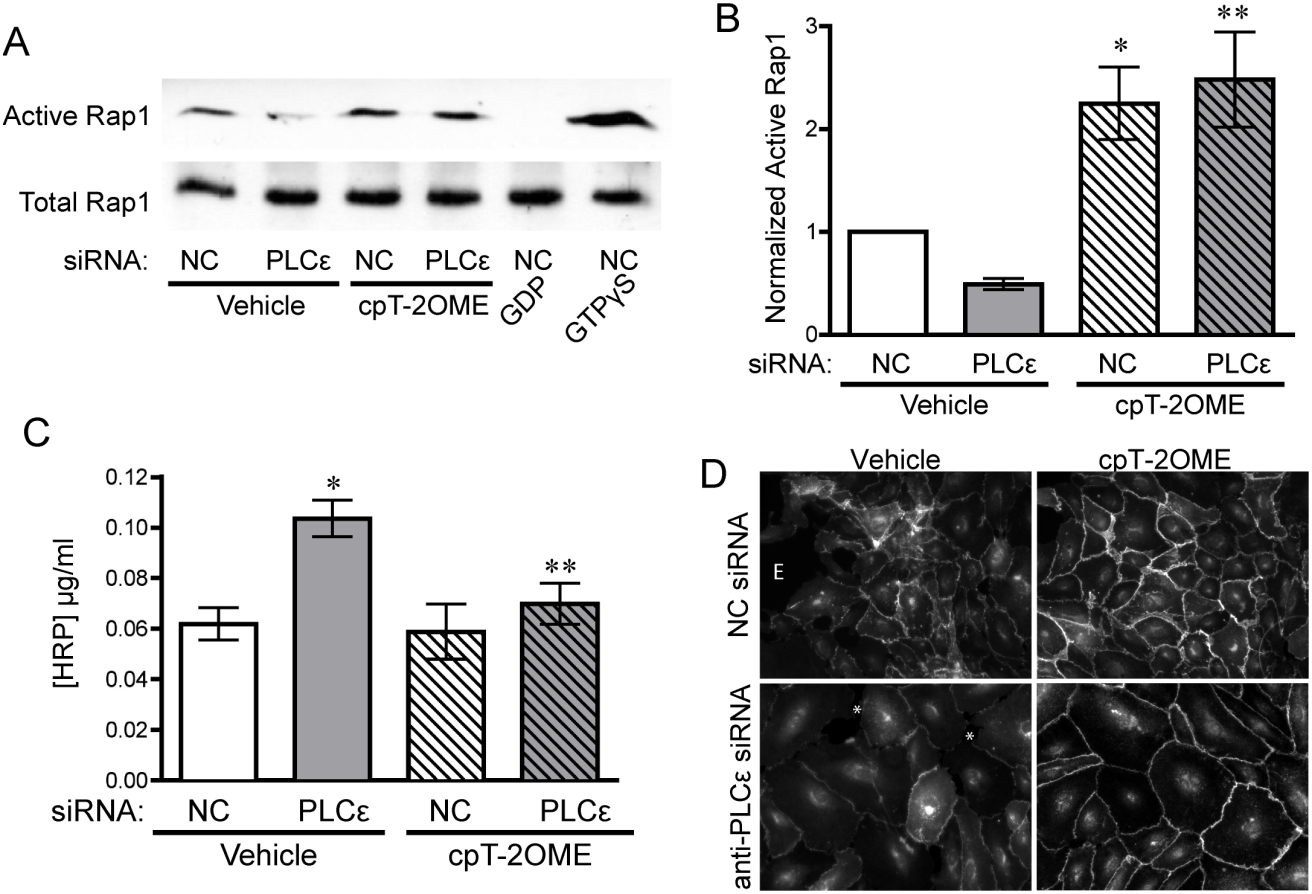
PLCε depletion leads to decreased basal Rap1 activity, which drives monolayer leak. **A**) Active Rap1 pulldown assay from lysates infected with negative control (NC) or PLCε siRNA ± 1μM 8-pCPT-2′-O-Me-cAMP-AM (cPT-2OMe), 1mM GDP, or 100μM GTPγS. Blots are representative, n = 7. **B**) Densitometric quantification of blots in (A). Data shown are active Rap1 normalized to total Rap1 ± SEM, n = 7, p = 0.0001 by ANOVA, *p<0.05 vs. NC infected cells by post-hoc test, and **p<0.05 vs. PLCε siRNA infected cells. All blots used for quantification can be found in S6 Fig. **C**) HRP leak through HPAEC monolayer infected with NC or PLCε siRNA ± 1μM 8-pCPT-2′-O-Me-cAMP-AM. Data shown are the mean HRP concentration, ± SEM. N = 5, p = 0.0022 by ANOVA, *p<0.001 by post-hoc test vs. NC infected cells and **p<0.05 vs. PLCε siRNA infected cells. **D**) VE-cadherin stained HPAEC infected with negative control or PLCε siRNA ±1μM 8-pCPT-2′-O-Me-cAMP-AM (cPT-2Ome). * indicates gaps occurring between endothelial cells. E- edge of monolayer. Images are representative of 3 separate experiments.

The finding that activation of Rap1 was able to reverse the increased RhoA activity in PLCε-deficient cells suggested that basal Rap1 activity is reduced in PLCε-deficient cells, similar to what has been reported in PLCε knockout mouse hearts[4]. Knockdown of PLCε in HPAEC decreased active Rap1 levels 50% compared to control cells, which could be restored by 8-pCPT-2′-O-Me-cAMP-AM stimulation (Fig 3A and 3B). Correspondingly, treatment of PLCε-deficient endothelial cells with 8-pCPT-2′-O-Me-cAMP-AM was also able to prevent increased monolayer leak (Fig 3C), indicating that the loss of Rap1 activity was responsible for the appearance of these phenotypes. As Rap1 activation stabilizes junctional VE-cadherin through its effector KRIT1[19], we also examined VE-cadherin expression and localization. Knockdown of PLCε did not significantly alter VE-cadherin expression (S2 Fig) but reduced the intensity of VE-cadherin staining at cell junctions compared to control siRNA infected cells (Fig 3D and S2 Fig). This decrease was reversed following treatment with 8-pCPT-2′-O-Me-cAMP-AM, suggesting that loss of Rap1 activity in PLCε deficient cells leads to the reduction in VE-cadherin at sites of cell-cell contact.

### The CDC25 and lipase activity of PLCε maintain active Rap1 levels and limit monolayer leak and stress fiber formation

**Fig 4:**
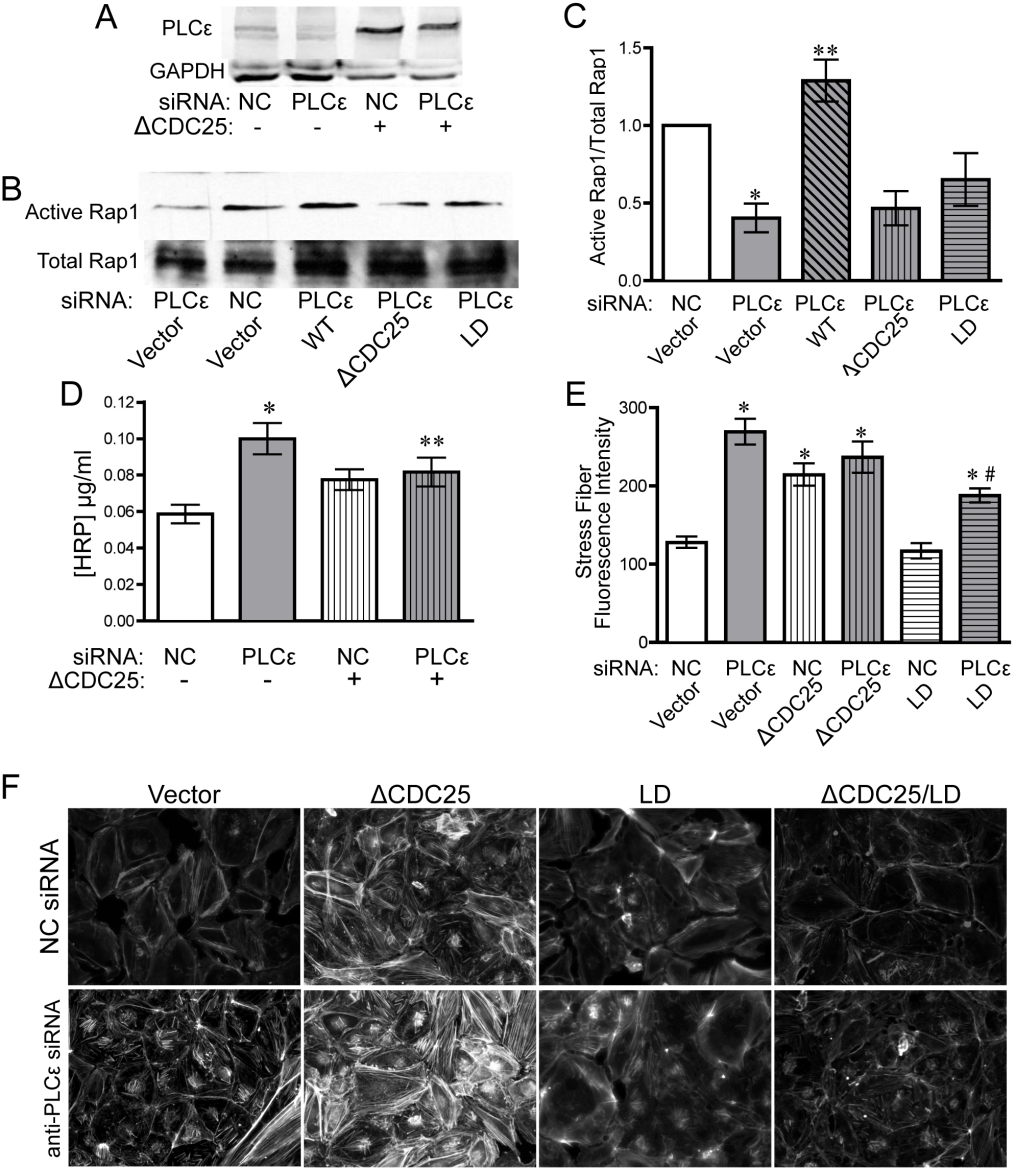
The CDC25 domain of PLCε maintains monolayer integrity and basal Rap1 activity. **A**) PLCε expression in HPAEC infected with NC or PLCε siRNA ± PLCε ΔCDC25. Blots are representative, n = 4. **B**) Active Rap1 pulldown assay from lysates infected with negative control or PLCε siRNA ± siRNA resistant PLCε (WT), PLCε ΔCDC25, or PLCε lipase dead (LD). Blots are representative, n = 5. **C**) Densitometric quantification of blots in (B). Data shown are active Rap1 normalized to total Rap1 ± SEM, n = 5, p = 0.0001 by ANOVA, *p<0.01 vs. NC infected cells by post-hoc test, and **p<0.001 vs. PLCε siRNA infected cells. All blots used for quantification can be found in S7 Fig. **D**) HRP leak through HPAEC monolayer infected with negative control (NC) or PLCε siRNA ± PLCε ΔCDC25 (ΔCDC25). Data shown are the mean HRP concentration, ± SEM. n = 17, p<0.0001 by ANOVA, *p<0.001 by post-hoc test vs. NC infected cells and **p<0.05 vs. NC infected cells. **E**) Fluorescent intensity quantification of actin staining in (F). n≥40 cells from 10 fields of view, p<0.0001 by ANOVA, *p<0.001 by post-hoc test vs. NC infected cells. #p<0.001 vs. PLCε siRNA infected cells. **F**) Epifluorescence images of rhodamine-phalloidin stained HPAEC infected with negative control or PLCε siRNA ± ΔCDC25, LD, or ΔCDC25/LD. Images are representative of 3 separate experiments.

The CDC25 domain of PLCε has been reported to function as a Rap1 GEF, leading to increased Rap1 activity when overexpressed with Rap1 in COS-7 cells, as well as increased GDP release in an in vitro GEF assay[3]. To confirm that the CDC25 domain of PLCε was responsible for maintaining basal Rap1-GTP levels in our system, and determine whether this domain is necessary for stabilizing endothelial barrier function, we expressed a PLCε mutant lacking the CDC25 GEF domain (ΔCDC25) in PLCε-deficient HPAEC (Fig 4A). Reconstitution of PLCε-deficient HPAEC with the ΔCDC25 mutant was not able to rescue decreased Rap1-GTP levels (Fig 4B and 4C) and was unable to reverse the increase in monolayer leak or the changes in actin cytoskeletal morphology (Fig 4D–4F). While it appeared that ΔCDC25 increased barrier function slightly, there was no statistical difference between PLCε siRNA transfected cells with or without co-expression of ΔCDC25 (Fig 4D). In addition, ΔCDC25 appeared to have a slight dominant negative effect in control cells, but that also did not reach statistical significance. Rap1 can also interact with the C-terminal RA2 domain of PLCε [8]. However, a PLCε construct with a binding-defective RA2 domain[5] was able to efficiently rescue monolayer leak and had no effect on Rap1-GTP levels (S3 Fig).

**Fig 5:**
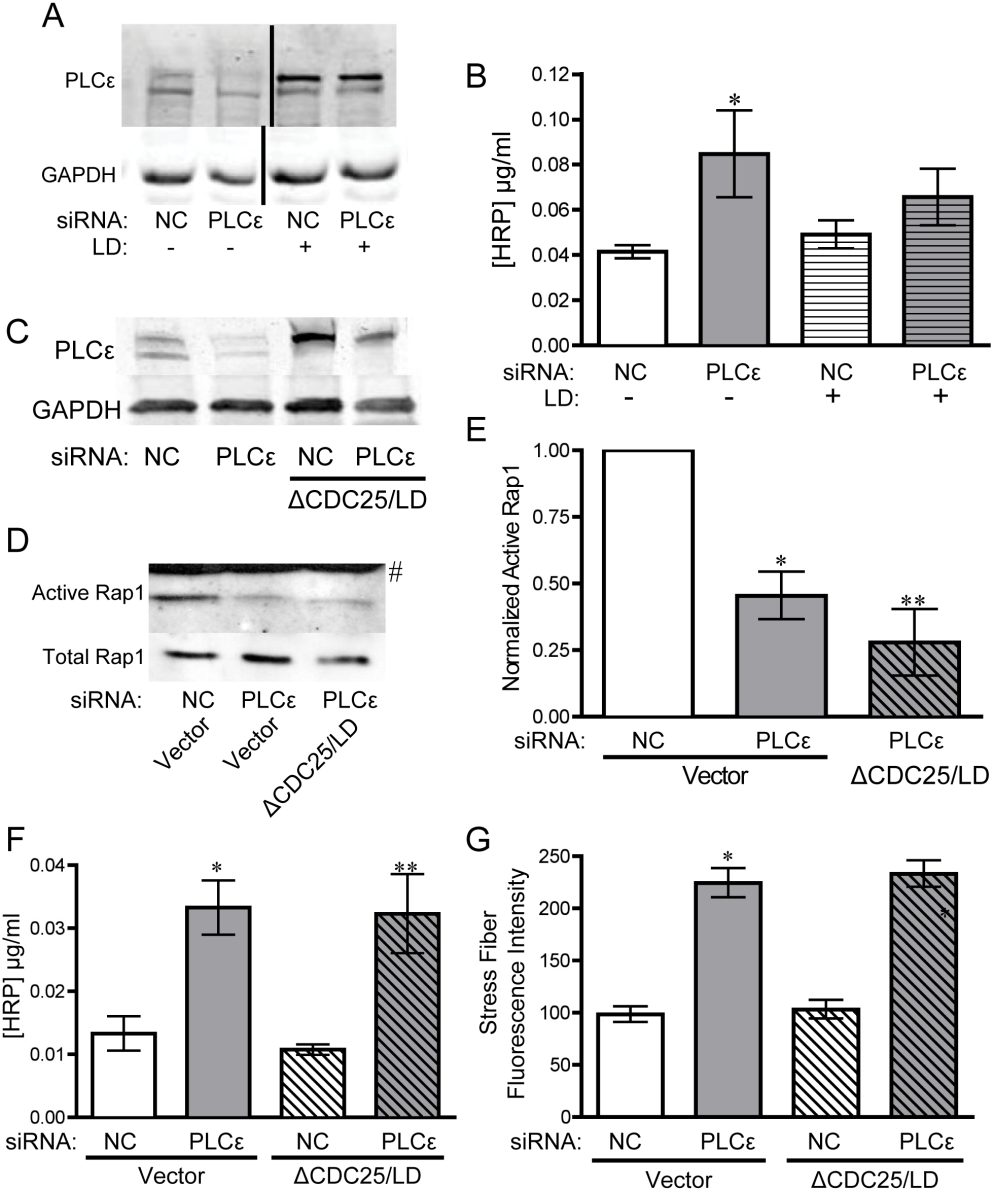
PLCε lacking the CDC25 domain and lipase activity cannot rescue monolayer integrity and decreased active Rap1 levels. **A**) PLCε expression in HPAEC infected with NC or PLCε siRNA ± PLCε lipase dead. Blots are representative. **B**) HRP leak through an HPAEC monolayer infected with negative control NC or PLCε siRNA ± PLCε lipase dead (LD). Data shown are the mean HRP concentration, ± SEM. n = 6, p = 0.002 by ANOVA, *p<0.001 by post-hoc test vs. NC infected cells. **C**) PLCε expression in HPAEC infected with NC or PLCε siRNA ± ΔCDC25/LD. Blots are representative, n = 3. **D**) Active Rap1 pulldown assay from lysates infected with negative control or PLCε siRNA ± ΔCDC25/LD. Blots are representative, n = 3. **E**) Densitometric quantification of blots in (D). Data shown are active Rap1 normalized to total Rap1 ± SEM, n = 3, p = 0.0007 by ANOVA, *p<0.01 vs. NC infected cells by post-hoc test, and **p<0.001 vs. NC siRNA infected cells. # indicates band from non-specific secondary antibody. All blots used for quantification can be found in S8 Fig. **F**) HRP leak through HPAEC monolayer infected with negative control (NC) or PLCε siRNA ± PLCε ΔCDC25/lipase dead (ΔCDC25/LD). Data shown are the mean HRP concentration, ± SEM. n = 9, p<0.0001 by ANOVA, *p<0.001 by post-hoc test vs. NC infected cells. **G**) Fluorescent intensity quantification of images of NC or PLCε siRNA infected cells ± ΔCDC25/LD. n≥40 cells from 10 fields of view, p<0.0001 by ANOVA, *p<0.001 by post-hoc test vs. NC infected cells.

As an additional control, we re-expressed a PLCε mutant lacking lipase activity (LD, Fig 5A). Unexpectedly, the lipase dead mutant also could not fully rescue monolayer leak (Fig 5B), despite equivalent protein expression, suggesting that both the GEF domain and lipase activity of PLCε are important for maintaining endothelial junctional stability. PLCε-LD transfected cells also exhibited a decrease in Rap-GTP and abnormal stress fiber morphology (Fig 4B, 4C, 4E and 4F), which corresponded with the impairment of barrier function. Because both the ΔCDC25 mutant and the lipase dead mutant appeared to partially rescue barrier function, we elected to make a PLCε mutant lacking both the CDC25 domain and lipase activity (ΔCDC25/LD, Fig 5C). This double mutant completely lacked any ability to rescue Rap1 GTP levels (Fig 5D and 5E), monolayer leak (Fig 5F), or stress fiber formation (Figs 4F and 5G) in PLCε-deficient HPAEC, lending extra support to the idea that both of these domains are involved in the regulation of Rap1 activity and maintenance of endothelial cell-cell contacts.

### PLCε lies upstream of KRIT1 signaling, through which it maintains barrier integrity

**Fig 6:**
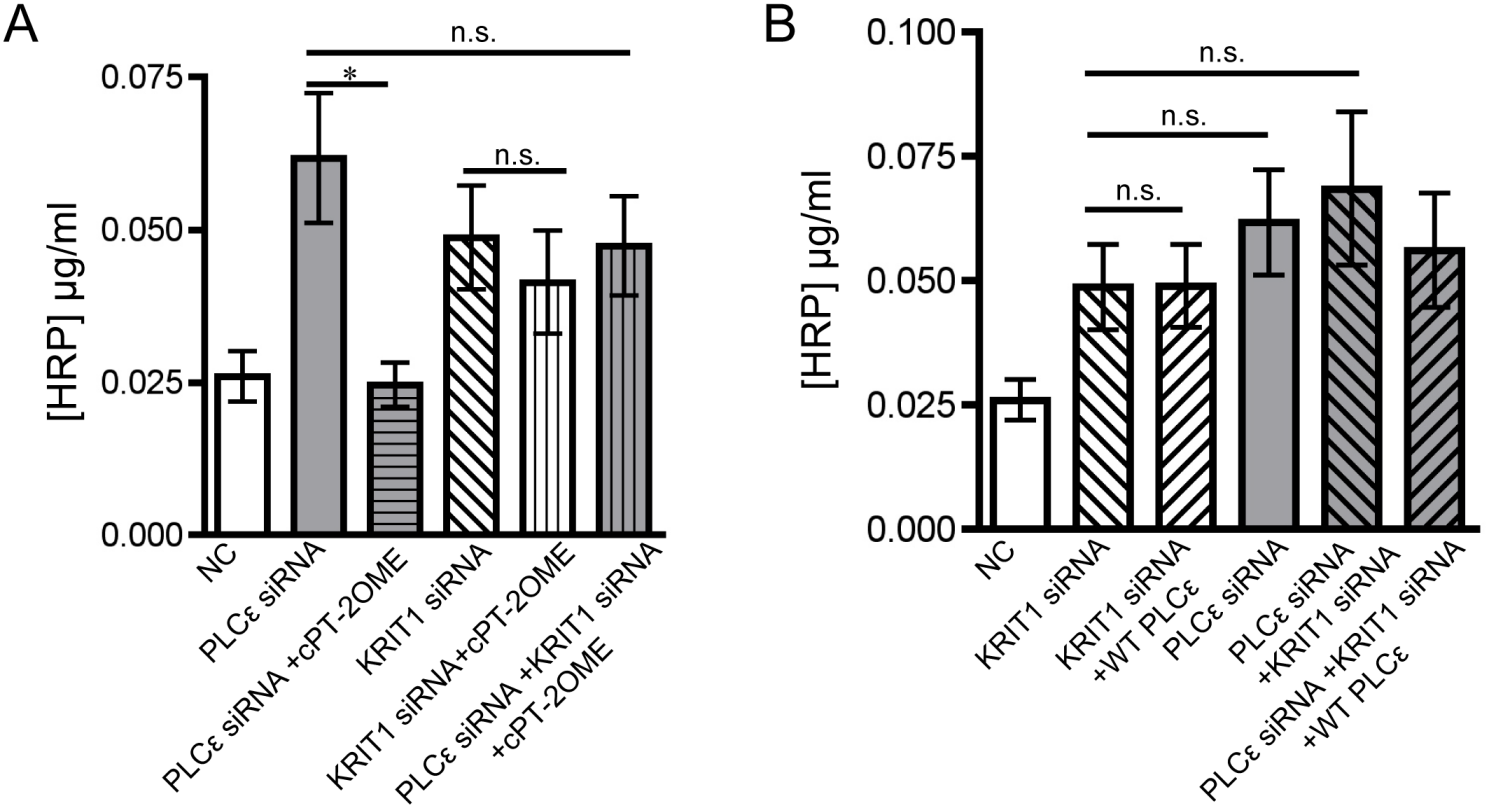
Loss of KRIT1 in PLCε depleted HPAEC prevents rescue by 8-pCPT-2′-O-Me-cAMP-AM and wild type PLCε. **A**) HRP leak through HPAEC monolayer infected with negative control (NC) or PLCε siRNA ± anti-KRIT1 siRNA or 1μM 8-pCPT-2′-O-Me-cAMP-AM (cPT-2OMe). Data shown are the mean HRP concentration, ± SEM. n = 9, p<0.0001 by ANOVA, *p<0.001 by post-hoc test vs. PLCε siRNA infected cells. n.s., non-significant. **B**) HRP leak through HPAEC monolayer infected with negative control (NC) or PLCε siRNA ± anti-KRIT1 siRNA or siRNA resistant PLCε (WT PLCε). Data shown are the mean HRP concentration, ± SEM. n = 9, p<0.0001 by ANOVA, n.s., non-significant.

The Rap1 effector, KRIT1, is vital for maintaining endothelial barrier stability, and acts as a endogenous inhibitor of junctional RhoA signaling[12,17,32]. Rap1-GTP promotes KRIT1 association with adherens junction complexes where it is thought to elicit its barrier protective effects(13). We hypothesized that PLCε-mediated activation of Rap1 would act through KRIT1 to stabilize the endothelial barrier. Knockdown of KRIT1 in PLCε-deficient endothelial cells (S4 Fig) did not have an additive effect on monolayer leak, but prevented rescue by Epac activation (Fig 6A). In addition, siRNA resistant WT PLCε could not rescue barrier function in PLCε-deficient cells in the absence of KRIT1 (Fig 6B). Together, these data provide evidence that PLCε lies upstream of KRIT1. In summary, our findings show that PLCε can promote endothelial barrier integrity by maintaining basal levels of Rap1-GTP/KRIT1 through its lipase and CDC25 GEF domain.

## Discussion

In this study, we have shown that PLCε is abundantly expressed in endothelial cells and its depletion leads to decreased barrier integrity (Fig 1) in arterial endothelial cells. The decrease in barrier integrity corresponds with increased Rho activity and subsequent stress fiber formation (Fig 2). Our evidence suggests that the loss of barrier function and Rho activation is due to reduced basal levels of Rap1, and subsequent KRIT1 signaling downstream, following PLCε knockdown (Figs 3 and 6). Surprisingly, both the CDC25 GEF domain and lipase activity of PLCε appear important for maintaining barrier function and active Rap1 levels (Figs 4 and 5). Together, our data suggest that PLCε activity functions to sustain basal levels of Rap1 activity required for maintenance of basal endothelial barrier function. This is the first evidence, to our knowledge, that a PLC isoform can have a barrier protective effect in the endothelium. This runs contrary to the known effects of other members of this family, such as PLCβ3, which have been reported to increase endothelial permeability[33]. We believe this unique capability is due to the presence of the CDC25 Rap GEF domain. This suggests that activation of PLCε may play a negative feedback role, limiting the loss of permeability in endothelial cells exposed to general PLC-activating signals.

While we fully expected that the CDC25 domain would be required, we were surprised to find that the lipase domain of PLCε was also required to fully restore barrier function in PLCε depleted cells. Lipase activity of other phospholipases has conventionally been associated with loss of endothelial barrier function[34–36]. Canonically, PLC lipase activity produces IP_3_, which leads to increased intracellular calcium and DAG which, together with calcium, activates protein kinase C (PKC). Both calcium and PKC have been identified as second messengers in pathways which increase endothelial permeability[37–41]. However, some have suggested that in vivo, PKC activity is required to maintain basal levels of permeability, and conversely is not involved in inflammation-induced permeability[38]. Recent work from our laboratory supports the idea that PLCε may signal primarily through PKC; PLCε activity results in sustained production of DAG from PI_4_P, rather than PIP_2_, and thus sustained PKC activation[21,42]. During revision of this paper, Bijli, et al published that loss of PLCε protects against vascular disruption and acute lung injury in response to lipopolysaccharide[43], suggesting that the role of PLCε in regulating inflammation-induced endothelial permeability may be distinct from its role in modulating baseline endothelial permeability. As activation of Rap1 by Epac can prevent the loss of endothelial barrier function due to thrombin-dependent activation of RhoA(11), it may be the case that PLCε acts through a Rap1-independent mechanism to regulate the endothelial response to inflammatory stimuli. Future experiments are necessary to explore the potential differences in signaling downstream of PLCε under inflammatory vs. unstimulated conditions, particularly regarding the importance of sustained PKC activation.

Alternatively, the requirement for the lipase domain could be explained by cross-talk between the lipase domain and the CDC25 domain. Recently, Dusaban et al proposed a positive feedback mechanism whereby Rap1 activation by the CDC25 domain promotes Rap1 association with RA2, and a subsequent increase in PLCε lipase activity[44]. While this feed-forward loop does not appear critical to endothelial barrier function, as a PLCε construct containing a function blocking mutation of the RA2 domain was able to rescue all endothelial phenotypes associated with PLCε depletion (S3 Fig), this mechanism does support the existence of autoregulation between different domains of PLCε. Clearly, further study is necessary to investigate how PLCε second messengers could promote/inhibit endothelial barrier stability, as well as determine under what conditions (i.e. subcellular/microdomain localization of PLCε) lipase activity positively or negatively affects endothelial barrier function.

Dusaban et al also observed that activation of PLCε leads to sustained Rap1 activation[44], which would likely have a major effect on cell adhesion in endothelial cells. As Rap1 is a key regulator of adherens junction stability and integrin activation, cell-cell and cell-extracellular matrix adhesion would likely be increased. Similarly, in neonatal rat ventricular myocytes, PLCε is vital for maintaining sustained Rap1 activation[4]. In myocytes, this leads to increased PKD activation, hypertrophic gene up-regulation[42], and in astrocytes and inflamed endothelial cells, to activation of pro-inflammatory NFκB signaling[43,45]. We can only speculate about the potential for PKD activation in our system, but as PKD is thought to exert primarily pro-inflammatory effects on the endothelium, we would expect PLCε activity in endothelial cells would promote, not protect against, loss of barrier function, as was seen under inflammatory conditions[43]. This suggests that the outcome of PLCε activity is likely highly dependent on cell type and the local signaling environment.

Lastly, our studies show that PLCε lies upstream of KRIT1 signaling. Previous studies have shown that the KRIT1-Rap1 interaction and subsequent increase in membrane associated KRIT1 is important for the ability of Rap1 to limit monolayer leak[14,15]. Our study provides further evidence that KRIT1 is a key Rap1 effector for maintaining endothelial barrier function,. However, other Rap1 effectors, Rasip and Radil, have been implicated in the stabilization of the endothelial barrier by Rap1[46,47], where they appear to have a similar function in blocking Rho activity as does KRIT1[17]. It is unknown whether Rasip/Radil are important Rap1 effectors downstream of PLCε activity, nor whether they act in parallel to KRIT1, or work synergistically. While our data clearly suggests that activation of the Rap1-KRIT1 signaling axis may be a widespread mechanism for regulating cell-cell contact, additional studies will be needed to asses a putative role for Rasip and Radil downstream of PLCε.

Lastly, we do note that PLCε knockout mice are viable and fertile[25], and exhibit none of the pathologies seen in KRIT1 or Rap1 loss-of-function mutants in mice and humans[48–53]. Thus PLCε -dependent activation of the Rap1-KRIT1 signaling axis is dispensable during development and in adulthood, suggesting that redundant mechanisms are in place to activate Rap1. However, conditional PLCε knockout models have shown significant differences with global knockout models. For example, global loss of PLCε potentiates cardiac hypertrophy[25] while deletion of PLCε in the heart limits it[42]. These divergent findings suggest that an endothelial specific PLCε knockout may be required to fully explore the endothelial role of PLCε.

In conclusion, our results have shown a novel role of PLCε in the maintenance of endothelial barrier function, via its CDC25 GEF domain and lipase activity, and subsequent up-regulation of Rap1 activity. Although the involvement of the lipase domain is surprising, these data point to a potential ability of this enzyme to promote barrier integrity through calcium or DAG dependent mechanisms, and also support the idea that the function of PLCε is cell type and context dependent. Given the thought-provoking role of PLCε in basal and inflammatory endothelial barrier function, these findings may stimulate future research into the role of PLCε in models of angiogenesis or vascular pathologies.

## Supporting Information

S1 Fig Knockdown of PLCε does not increase 150kDa FITC-dextran flux. 150 kDa FITC-dextran leak through an HPAEC monolayer infected with negative control (NC) ± 4U/ml thrombin or PLCε siRNA. Data shown are RFU ± SEM. n = 4.
(JPG)

S2 Fig Knockdown of PLCε reduces VE-cadherin junctional thickness. **A**) Western blot expression of VE-cadherin and GAPDH in HPAEC infected with infected with negative control (NC) or PLCε siRNA ± 1μM 8-pCPT-2′-O-Me-cAMP-AM (cPT-2OMe). Blots are representative, n = 3. **B**) Quantification of (A). Data shown are VE-cadherin normalized to GAPDH ± SEM. n = 3. **C**) Fluorescent intensity quantification of images of NC or PLCε siRNA infected cells ±1μM 8-pCPT-2′-O-Me-cAMP-AM (cPT-2OMe). n≥40 cells from 10 fields of view.
(JPG)

S3 Fig The RA2 domain of PLCε does not regulate monolayer integrity, basal Rap1 activity or stress fiber formation. **A**) HRP leak through HPAEC monolayer infected with negative control (NC) or PLCε siRNA ± PLCε K2150E. Data shown are the mean HRP concentration, ± SEM. n = 4. **B**) PLCε expression in HPAEC infected with NC or PLCε siRNA ± PLCε K2150E (RA2 binding-deficient). Blots are representative, n = 4. **C**) Densitometric quantification of active Rap1 pulldown assay from lysates infected with negative control or PLCε siRNA ± PLCε K2150E. Data shown are active Rap1 normalized to total Rap1 ± SEM, n = 4. **D**) Fluorescent intensity quantification of images of NC or PLCε siRNA infected cells ± PLCε K2150E. n≥40 cells from 10 fields of view.
(JPG)

S4 Fig Representative blot of KRIT1-depletion in Fig 6. Immunoprecipitation of KRIT1 from NC or PLCε siRNA infected lysates ± anti-KRIT1 siRNA, 1μM 8-pCPT-2′-O-Me-cAMP-AM, or siRNA resistant PLCε (WT PLCε). Blots are representative, n = 3.
(JPG)

S5 Fig Western blots used for quantification in Fig 2A/2B. (JPG)

S6 Fig Western blots used for quantification in Fig 3A/3B. (JPG)

S7 Fig Western blots used for quantification in Fig 4B/4C. (JPG)

S8 Fig Western blots used for quantification in Fig 5D/5E. (JPG)
