# Supplementary figures and images for: Phospholipase Cε Modulates Rap1 Activity and the Endothelial Barrier
Source: PLoS One. 2016 Sep 9;11(9):e0162338. doi: 10.1371/journal.pone.0162338 (PMC5017709; doi:10.1371/journal.pone.0162338)

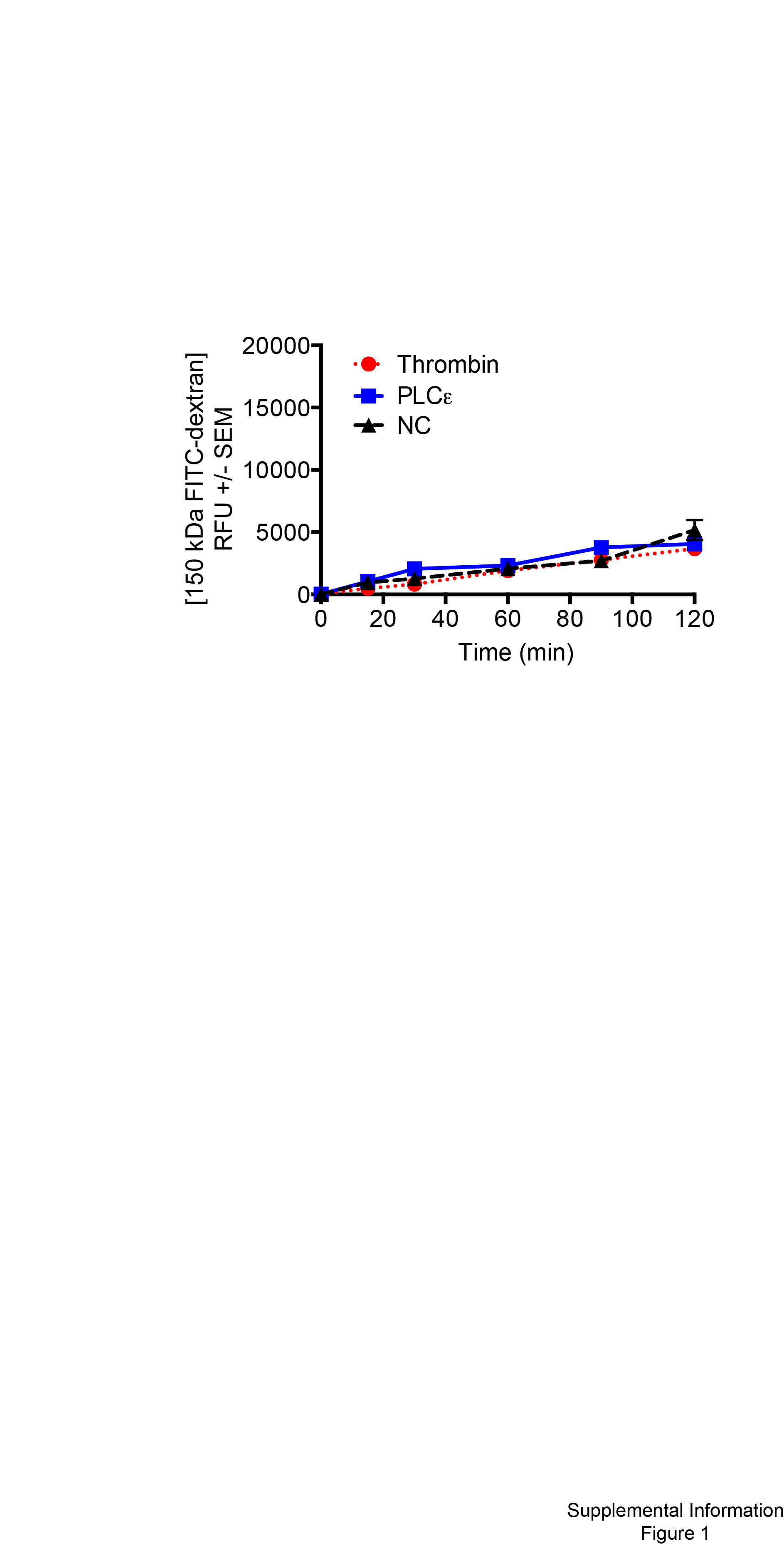

Supplement: S1 Fig — 150 kDa FITC-dextran leak through an HPAEC monolayer infected with negative control (NC) ± 4U/ml thrombin or PLCε siRNA. Data shown are RFU ± SEM. n = 4. (JPG) [file pone.0162338.s001.jpg]

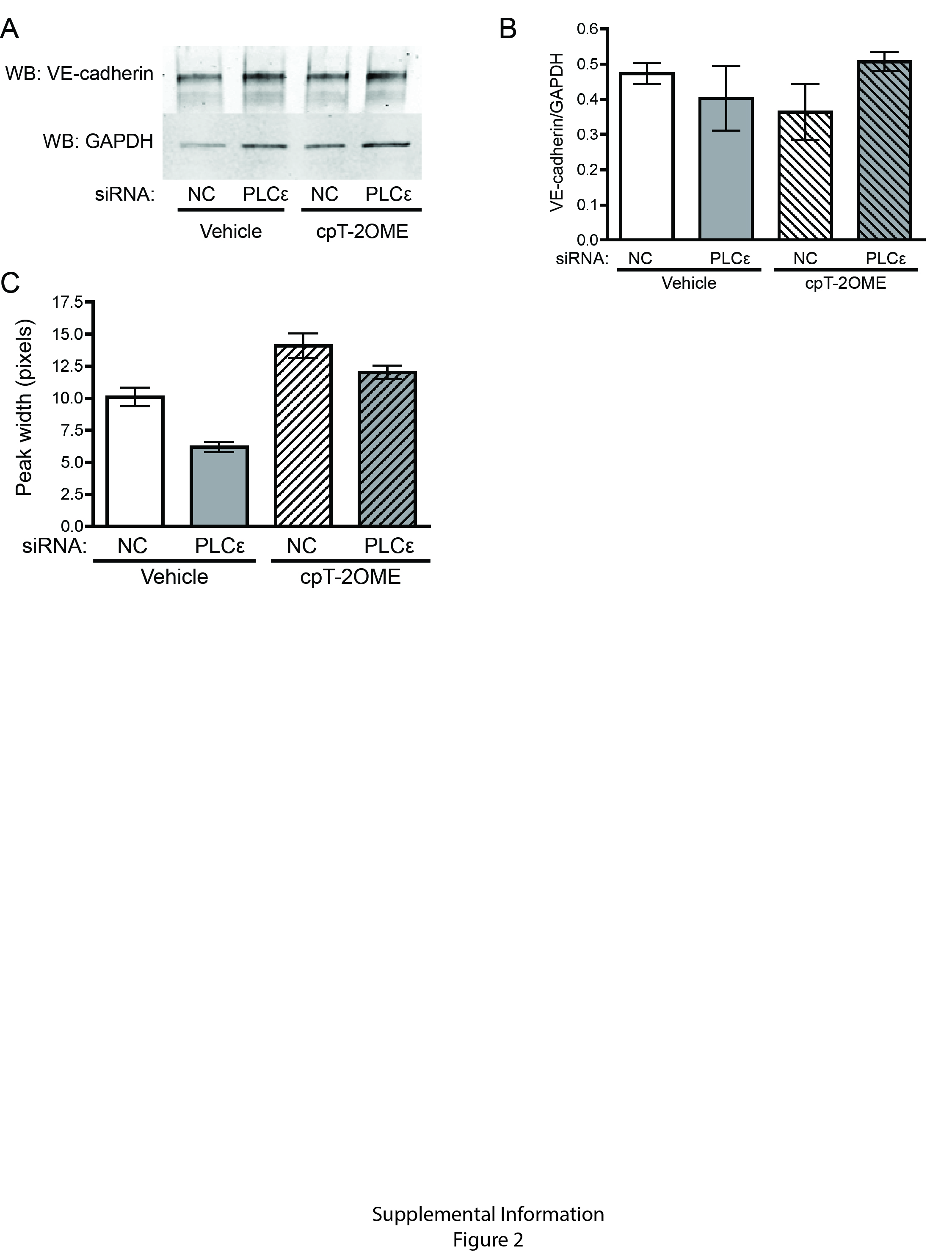

Supplement: S2 Fig — A) Western blot expression of VE-cadherin and GAPDH in HPAEC infected with infected with negative control (NC) or PLCε siRNA ± 1μM 8-pCPT-2′-O-Me-cAMP-AM (cPT-2OMe). Blots are representative, n = 3. B) Quantification of (A). Data shown are VE-cadherin normalized to GAPDH ± SEM. n = 3. C) Fluorescent intensity quantification of images of NC or PLCε siRNA infected cells ±1μM 8-pCPT-2′-O-Me-cAMP-AM (cPT-2OMe). n≥40 cells from 10 fields of view. (JPG) [file pone.0162338.s002.jpg]

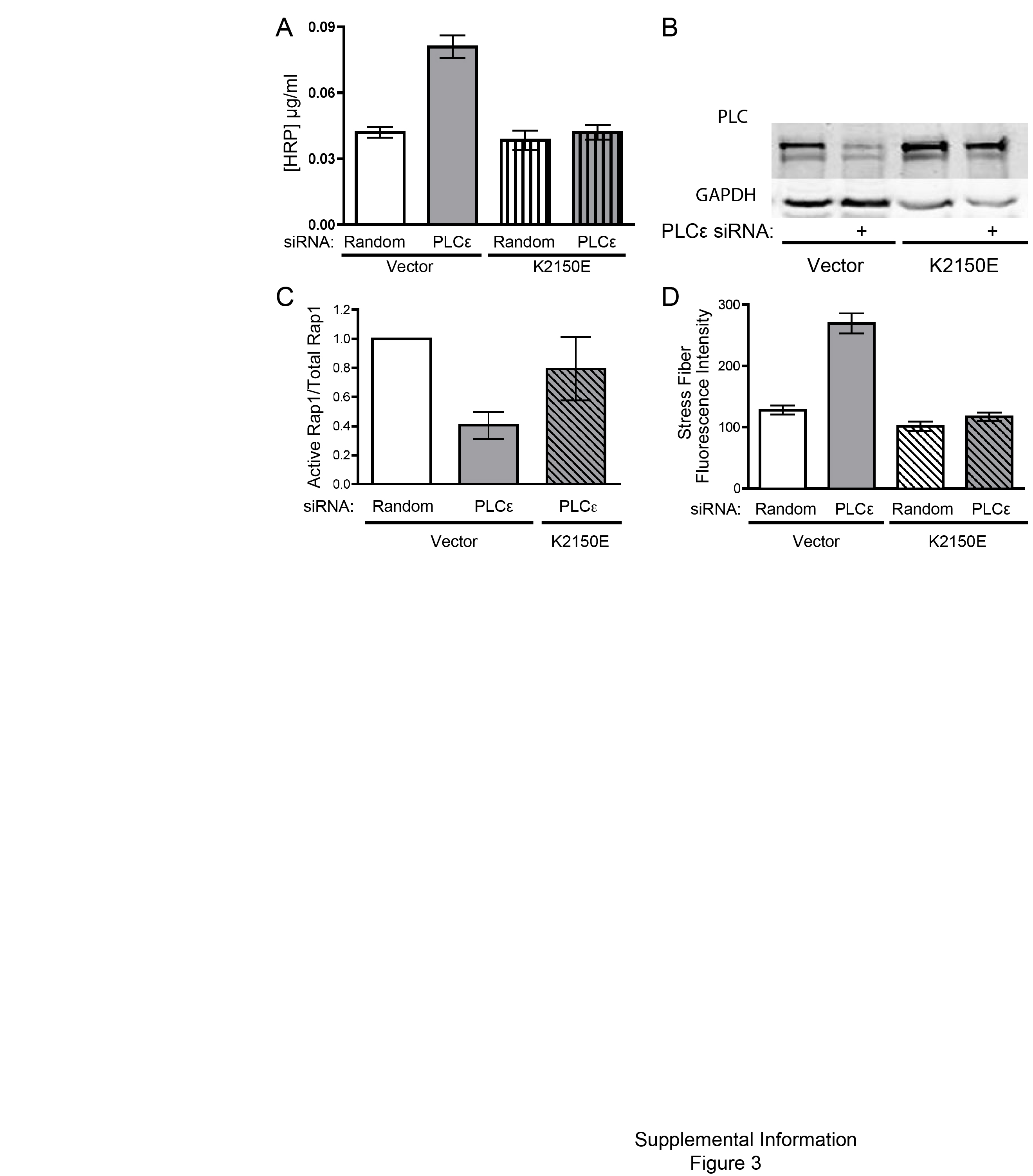

Supplement: S3 Fig — A) HRP leak through HPAEC monolayer infected with negative control (NC) or PLCε siRNA ± PLCε K2150E. Data shown are the mean HRP concentration, ± SEM. n = 4. B) PLCε expression in HPAEC infected with NC or PLCε siRNA ± PLCε K2150E (RA2 binding-deficient). Blots are representative, n = 4. C) Densitometric quantification of active Rap1 pulldown assay from lysates infected with negative control or PLCε siRNA ± PLCε K2150E. Data shown are active Rap1 normalized to total Rap1 ± SEM, n = 4. D) Fluorescent intensity quantification of images of NC or PLCε siRNA infected cells ± PLCε K2150E. n≥40 cells from 10 fields of view. (JPG) [file pone.0162338.s003.jpg]

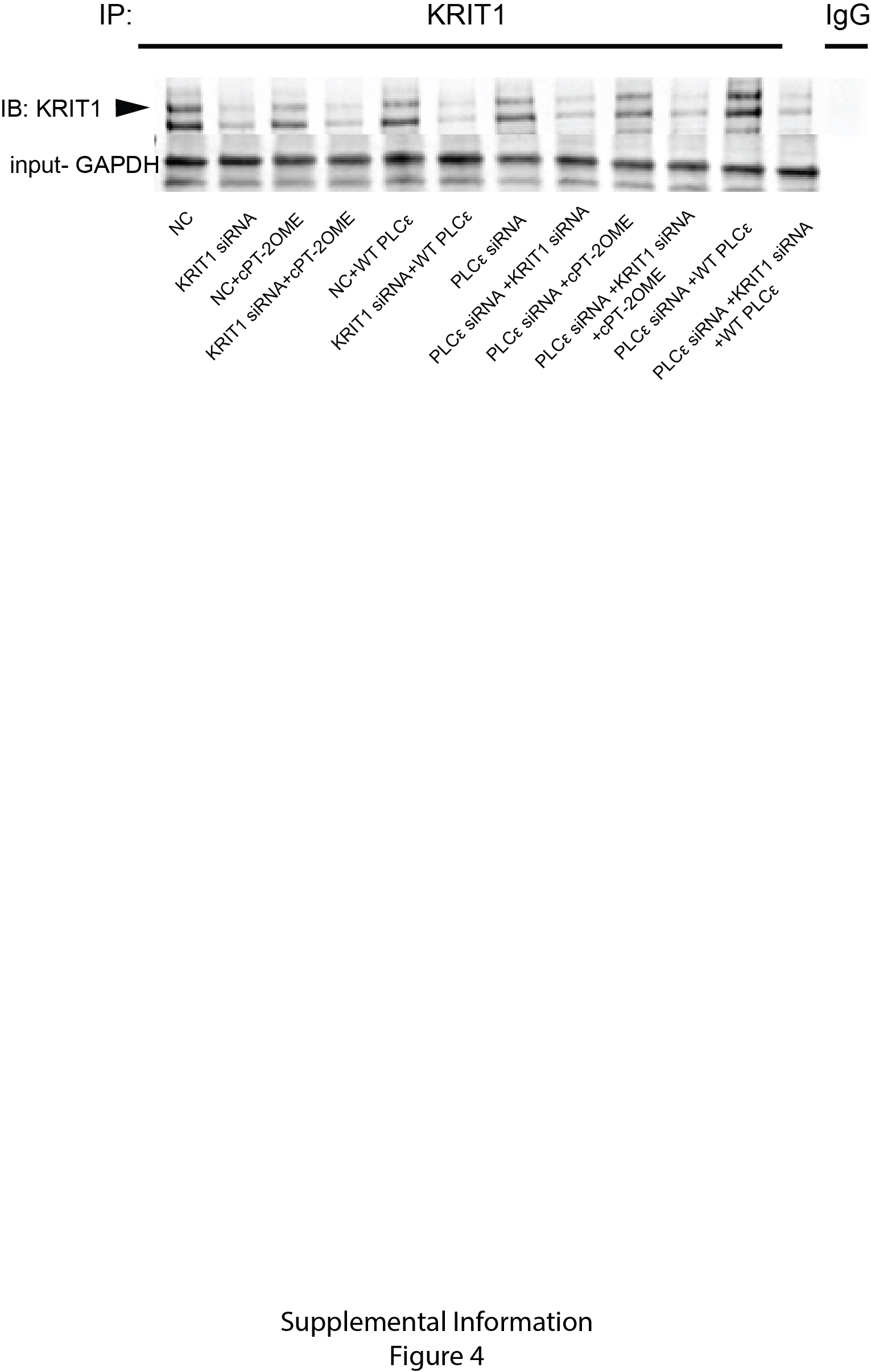

Supplement: S4 Fig — Immunoprecipitation of KRIT1 from NC or PLCε siRNA infected lysates ± anti-KRIT1 siRNA, 1μM 8-pCPT-2′-O-Me-cAMP-AM, or siRNA resistant PLCε (WT PLCε). Blots are representative, n = 3. (JPG) [file pone.0162338.s004.jpg]

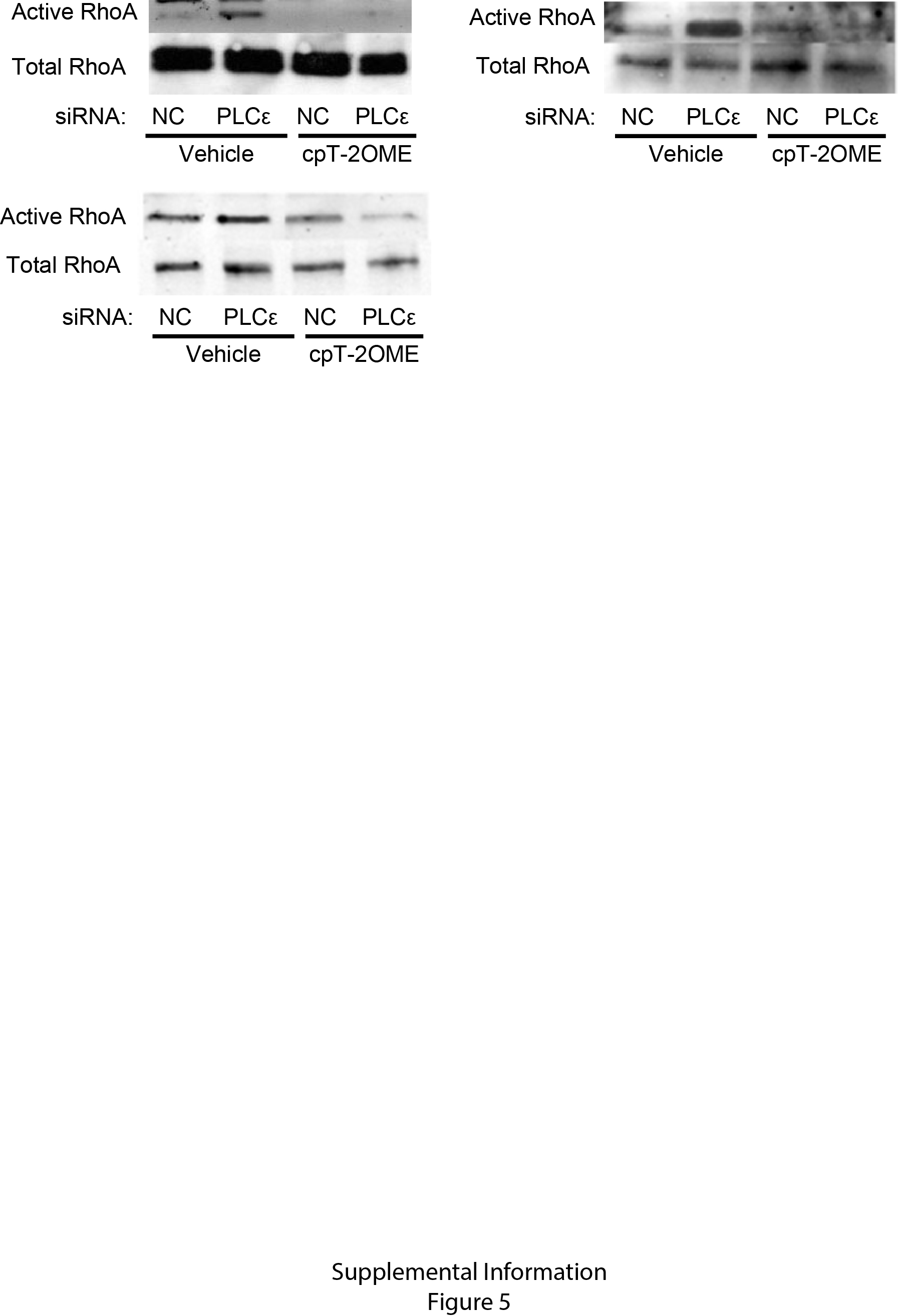

Supplement: S5 Fig — (JPG) [file pone.0162338.s005.jpg]

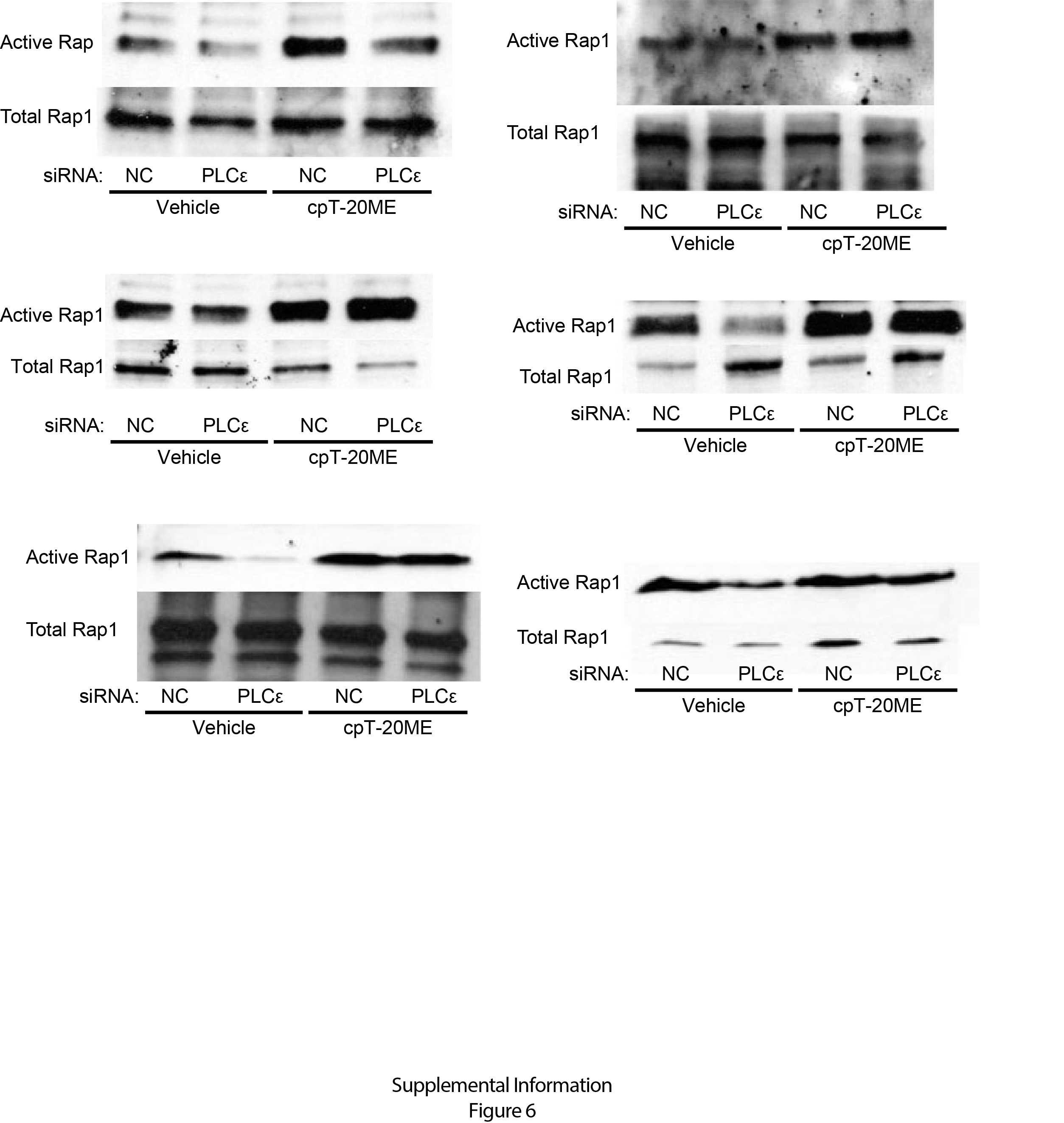

Supplement: S6 Fig — (JPG) [file pone.0162338.s006.jpg]

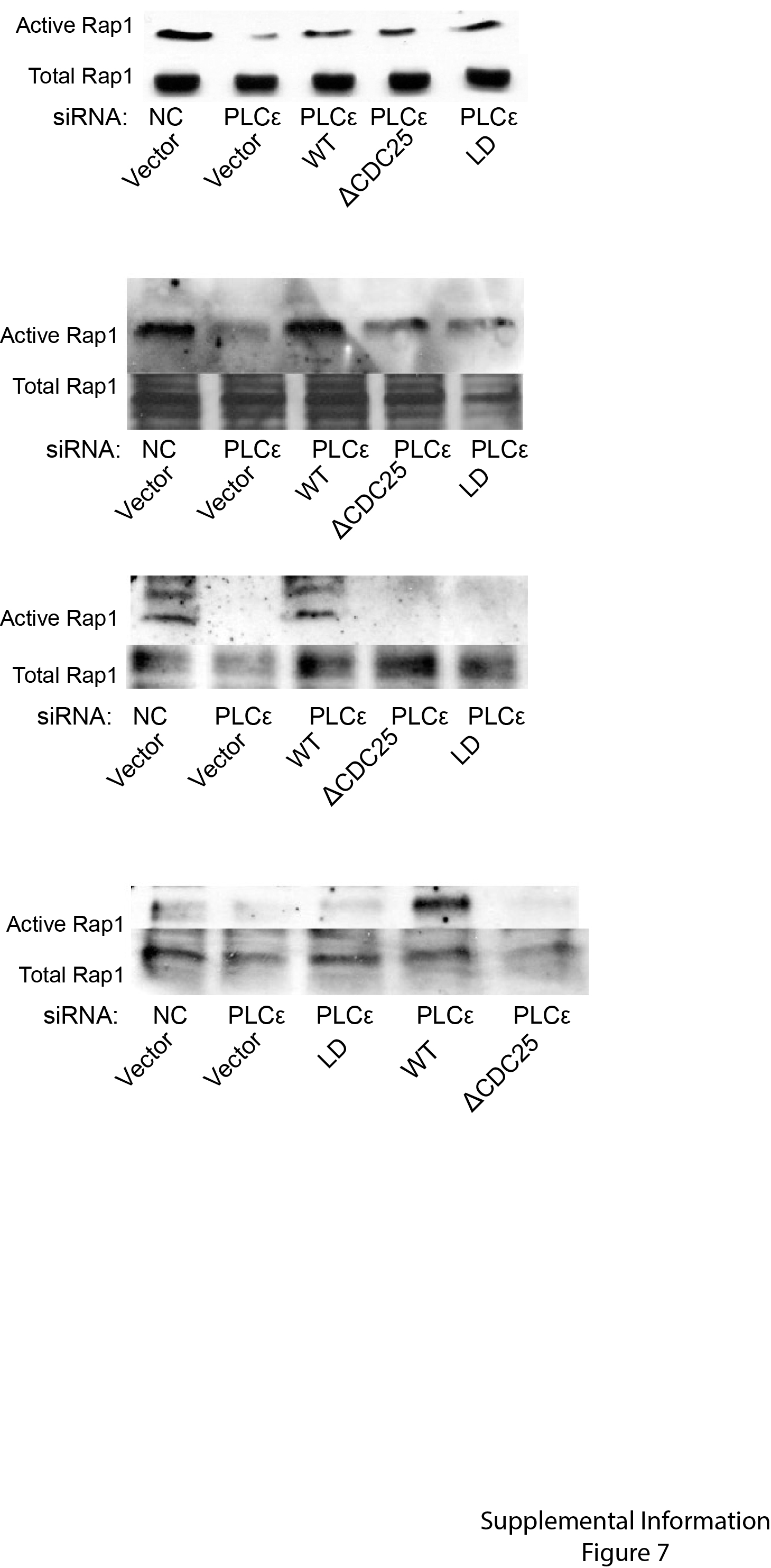

Supplement: S7 Fig — (JPG) [file pone.0162338.s007.jpg]

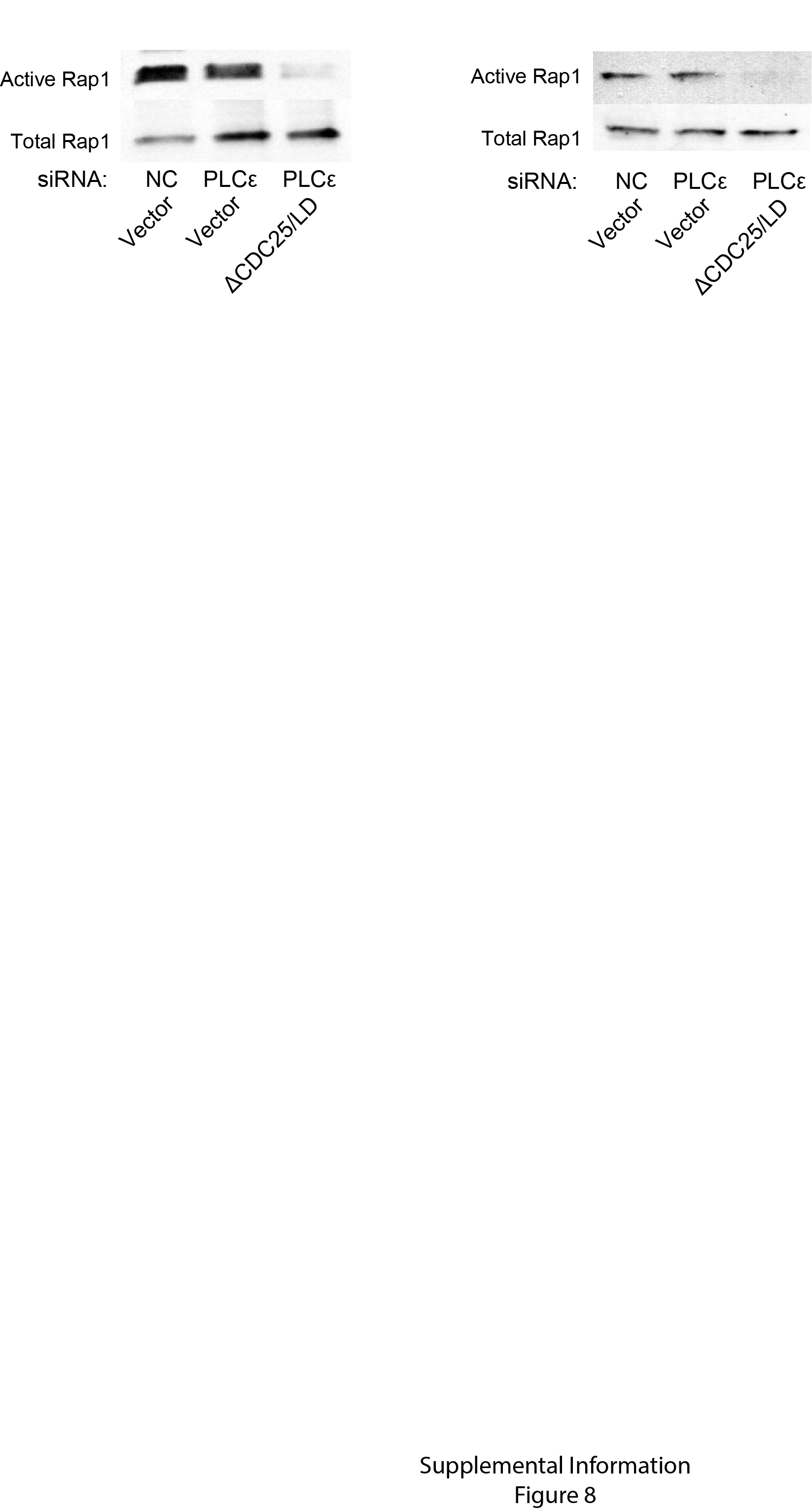

Supplement: S8 Fig — (JPG) [file pone.0162338.s008.jpg]
